# Dual-emitting quantum dot nanohybrid for imaging of latent fingerprints: simultaneous identification of individuals and traffic light-type visualization of TNT†
†Electronic supplementary information (ESI) available. See DOI: 10.1039/c5sc01497b


**DOI:** 10.1039/c5sc01497b

**Published:** 2015-05-21

**Authors:** Peng Wu, Chaoying Xu, Xiandeng Hou, Jing-Juan Xu, Hong-Yuan Chen

**Affiliations:** a State Key Laboratory of Analytical Chemistry for Life Science and Collaborative Innovation Center of Chemistry for Life Sciences , School of Chemistry and Chemical Engineering , Nanjing University , Nanjing 210093 , China . Email: xujj@nju.edu.cn ; Email: hychen@nju.edu.cn; b Analytical & Testing Center , Sichuan University , Chengdu 610064 , China

## Abstract

Fingerprints are a unique characteristic of an individual. Recently, it has been realized that fingerprints carry more information about individuals than just their identity, for example, they may identify potential addicts and terrorists carrying explosives. Therefore, the development of imaging moieties capable of both fingerprint staining and drug/explosive visualization is of significant importance for forensic chemistry. Here we developed a nanohybrid comprising green- and red-emitting QDs for simultaneous fingerprint imaging and TNT visualization in fingerprints. The red-emitting Cu-doped ZnCdS (Cu–ZnCdS) QDs were embedded into silica nanoparticles and the green-emitting ZnCdS QDs were anchored onto the surface of the silica nanoparticles and further functionalized with polyallylamine (PAA). Both components of the nanohybrid, *i.e.*, the PAA-functionalized green QDs and red QD-doped silica nanoparticles, could be explored for fingerprint imaging. Due to the formation of a Meisenheimer complex between TNT and PAA, the green-emitting QDs could be quenched by TNT, meanwhile the red-emitting QDs were inert. Therefore, the nanohybrid exhibited a traffic light-type fluorescence color change (green-yellow-red) to TNT concentration in the range of 40–400 μM. This method is promising for potential applications in security-screening needs in public areas such as airports and train stations.

## Abstract 2

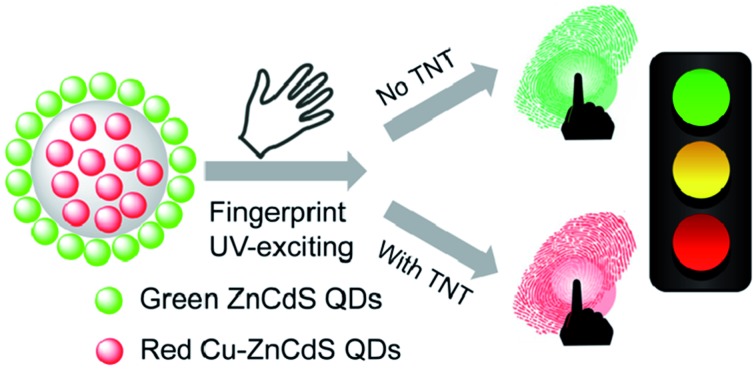
A nanohybrid was employed for fingerprint imaging that was capable of simultaneous identification of individuals and TNT visualization in a “traffic-light” manner.

## Introduction

Fingerprints are unique and invariable to an individual and hence are important for individual credentials, access control, and other forensic investigations, and are the cornerstone of forensic evidence. When a fingertip contacts a surface, residues are usually transferred to the substrate, thus leaving a latent fingerprint (LFP). As LFPs are invisible to the naked eye, they require either physical or chemical enhancement in order to make them visible and suitable for identification.^1^–^4^ Like many other scientific areas, forensic science is currently also beginning to embrace nanotechnology.^5^–^7^ This is particularly true for LFP detection for which photoluminescent nanoparticles (particularly quantum dots) have been a subject of intensive research efforts for more than a decade.^8^–^14^

Currently, fingerprints are very useful when police or other security agencies are able to obtain a positive match with those prints present in databases. Recently, it has been realized that fingerprints carry more information about individuals than just their identity,^15^–^22^ which adds new and challenging tasks and also opportunities for the forensic research community. For example, it is known that sweat contains a variety of metabolites of clinical significance. The Russell group thus pioneered the use of “intelligent” fingerprinting for simultaneous identification of drug metabolites and individuals.^15^ A nanoplasmonic imaging technique was developed for LFP imaging and identification of the abuse of illicit drugs.^20^ Apart from the potential of LFPs to serve as “samples” for use in medical diagnosis or identification of addicts, there is also substantial anti-terrorism interest in the use of LFPs for the screening of individuals in public areas such as airports and train stations.^23^ Terrorists carrying explosives may be experts in disguising themselves, but explosive traces left on their hands cannot be easily cleaned. Therefore, a facile and fast LFP imaging method for simultaneous identification of individuals and screening of explosive carriers is highly desired for police or security agencies.

Herein, we present a dual-emitting quantum dot (QD) nanohybrid for LFP imaging that is capable of simultaneous individual identification and TNT visualization. Green-emitting ZnCdS QDs and red-emitting Cu-doped ZnCdS (Cu–ZnCdS) QDs were employed in this work because of the superior optical properties of QDs.^24^–^27^ Both green- and red-emitting QDs could be successfully used in LFP staining (individual identification), but their responses to TNT were not sensitive enough for the naked eye. After assembling these two types of QDs with silica (nanohybrid of red QDs@silica@green QDs), the green fluorescence was quenched by TNT, while the red fluorescence was inert. Therefore, the developed nanohybrid exhibited a ratiometric response to TNT. The use of dual-emitting QDs for ratiometric sensing has been demonstrated to be truly appealing,^28^–^32^ but rare in fingerprint applications. Moreover, the change in the fluorescence color of the proposed nanohybrid was of “traffic light”-type (from green to red) after staining the LFPs with different TNT amounts, providing visual evidence for police or security agencies in screening tasks.

## Results and discussion

### Characterization of green- and red-emitting QDs and the nanohybrid

**Fig. 1:**
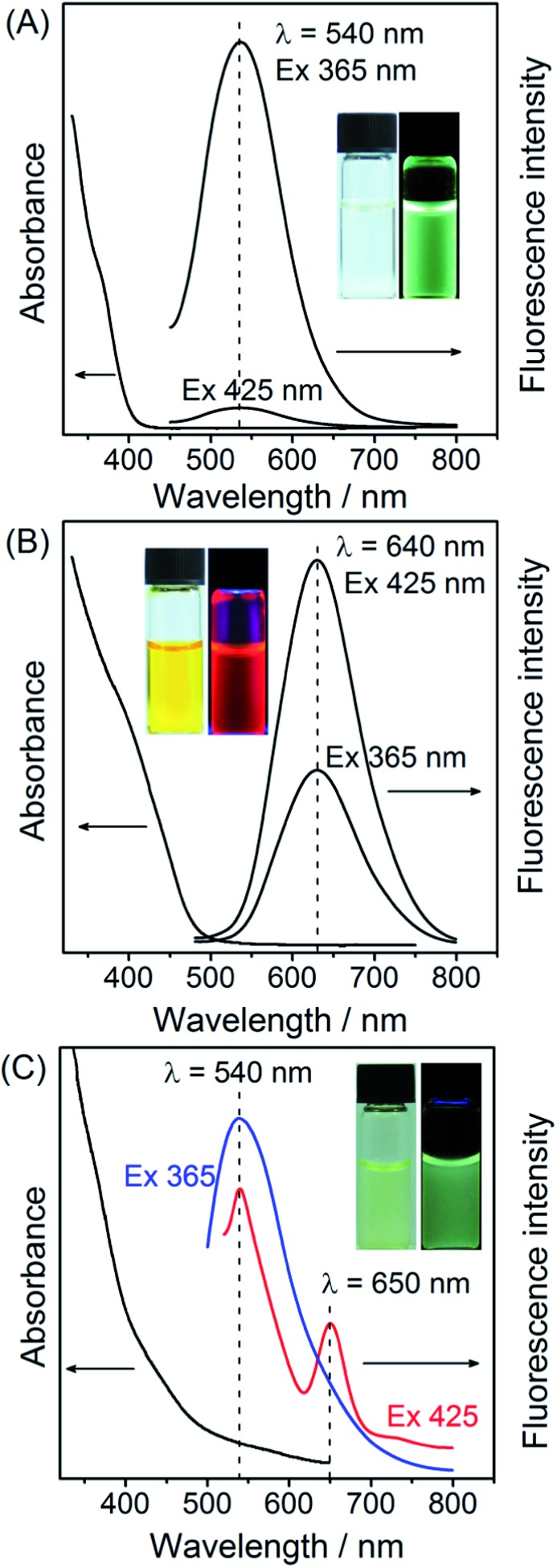
Spectroscopic characterization of dual-emitting QD nanohybrid: (A) UV-vis absorption and fluorescence emission spectra of green-emitting ZnCdS QDs; (B) UV-vis absorption and fluorescence emission spectra of red-emitting Cu-doped ZnCdS QDs; and (C) UV-vis absorption and fluorescence emission spectra of the red QD@silica@green QD nanohybrid. The photographs of the corresponding QDs or nanohybrid under daylight (left) and UV-excitation (365 nm, right) are shown in the insets.

In this research, ZnCdS QDs and Cu-doped ZnCdS QDs were employed to provide the green and red fluorescence colors, respectively. As shown in Fig. 1A and B, the UV-vis absorption spectra of the ZnCdS and Cu–ZnCdS QDs showed the characteristic absorption of the alloyed ZnCdS host (but different sizes, Fig. S1 of ESI†). For the ZnCdS QDs, a broad emission band centered at 540 nm was observed together with a green fluorescence color with UV-excitation (365 nm from the UV lamp, inset of Fig. 1A). This broad green emission band of the ZnCdS QDs is the characteristic defect-related emission as evidenced from its large Stokes shift. For the Cu–ZnCdS QDs, an emission band centered at 640 nm was obtained, showing a stable red fluorescence upon UV-excitation (365 nm from UV lamp, inset of Fig. 1B), similar to a previous report.^33^ The different fluorescence emission colors of the ZnCdS and Cu–ZnCdS QDs can be attributed to the different energy levels provided by doping as well as the different sizes of the QDs (Fig. S1 of ESI,† about 3 nm for ZnCdS QDs and 4 nm for Cu–ZnCdS QDs). Cu doping is a facile approach for adjusting the electronic and optical properties of QDs. Particularly for CdS or ZnCdS QDs, yellow to near-infrared dopant emission can be obtained.^25^,^34^–^36^ Elemental composition analysis with inductively coupled plasma optical emission spectroscopy (ICP-OES) confirmed that the content of Cu was about 1% (Table S1 of ESI†) and was in accordance with previous reports.^35^,^36^ Both green- and red-emitting QDs showed reasonable photostability (Fig. S2 of ESI†), which is crucial for imaging applications.

**Scheme 1:**
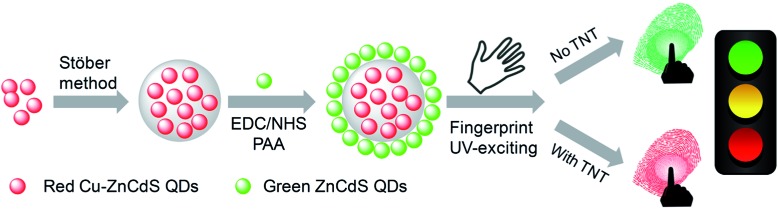
Schematic illustration of the preparation of the nanohybrid of green/red QDs for simultaneous LFP imaging and traffic light-type visualization of TNT.

The nanohybrid of red QDs@silica@green QDs for LFP imaging and TNT visualization was fabricated by first doping of red-emitting QDs into silica nanoparticles and then covalent bonding of green-emitting QDs to the surface of the resultant silica nanoparticles (Scheme 1 and ESI†).^28^ As shown in Fig. 1C, the UV-vis absorption of the nanohybrid comprised the absorption characteristics of both green-emitting and red-emitting QDs. The color of the nanohybrid suspension is light yellow (inset of Fig. 1B, also between that of green and red QDs). Upon excitation at 365 nm, the nanohybrid exhibited principally the emission profile of green QDs with a slight contribution from that of red QDs (Fig. 1C and S3 of ESI†), which is advantageous for the subsequent traffic light-type visualization of TNT (no TNT, green color). Overall, the nanohybrid showed a green color, but not as bright as that of the only green-emitting QDs (inset of Fig. 1C). When excited at 425 nm, which is closer to the maximum excitation of red QDs, the fluorescence intensity of the green-emitting QDs was significantly decreased; while that of the red-emitting QDs was enhanced about 2-fold. Therefore, well-resolved dual emission bands centered at 540 nm and 650 nm could thus be obtained from the nanohybrid (Fig. 1C), corresponding to fluorescence emission of the green-emitting and red-emitting QDs, respectively. After doping into silica, the fluorescence emission wavelength of the red-emitting QDs underwent about a 10 nm red shift, possibly because of the surface state change of the QDs during the incorporation into silica. The overall irregular emission profile of the nanohybrid was largely caused by the scattering from the silica nanoparticles. The photostability of the nanohybrid is also reasonably good for further imaging applications.

### Imaging of LFPs with green- and red-emitting QDs

**Fig. 2:**
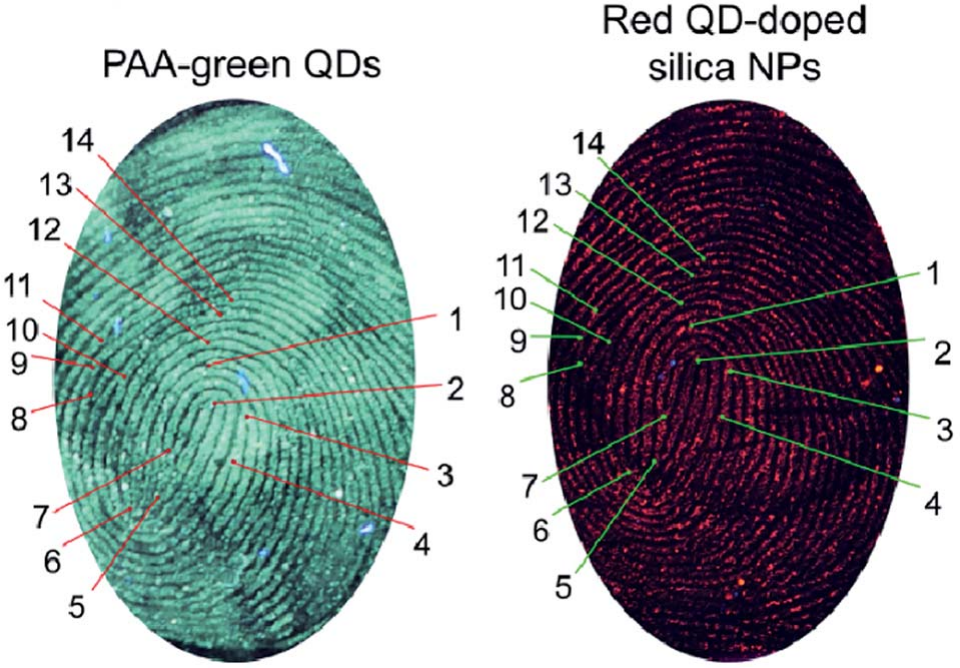
LFP images based on PAA–green QDs and red QD-doped silica nanoparticles. Some of the similar minutia points of the two fingerprints are labeled on the images. The fluorescence images were excited with a 365 nm UV lamp.

The excellent optical properties of QDs have been extensively explored for LFP imaging.^8^–^11^ Here, we first demonstrated that the two main components of the nanohybrid, namely the as-prepared polyallylamine (PAA)-functionalized green QDs and the red QD-doped silica nanoparticles, could be used for LFP imaging and individual identification. As shown in Fig. 2, both types of QDs could successfully stain LFPs. Without QD-staining, the LFPs are barely visible. The driving force for the staining of fingerprints with QDs and the nanohybrid may include electrostatic and also hydrophobic interactions.^2^,^9^ The sub-structures of LFPs in principle form the basis of the forensic identification of individuals. The LFP images stained with green and red QDs displayed well-resolved ridge flow and pattern configuration (level 1, Fig. 2). In addition, level 2 (ridge termination, bifurcation, and lake) characteristics of the LFP were also clearly observed (Fig. S5 of ESI†). Moreover, the LFP images obtained by these two types of QDs were generally the same (from the same volunteer), since the macro details such as pattern type, singular points, and friction ridge flow of the two images are similar. Additionally, a series of similar minutia points of the two images can be found (14 of which are labelled in Fig. 2), confirming the accuracy of using green and red QDs for LFP detection.

### Ratiometric fluorescence response of the nanohybrid to TNT

**Fig. 3:**
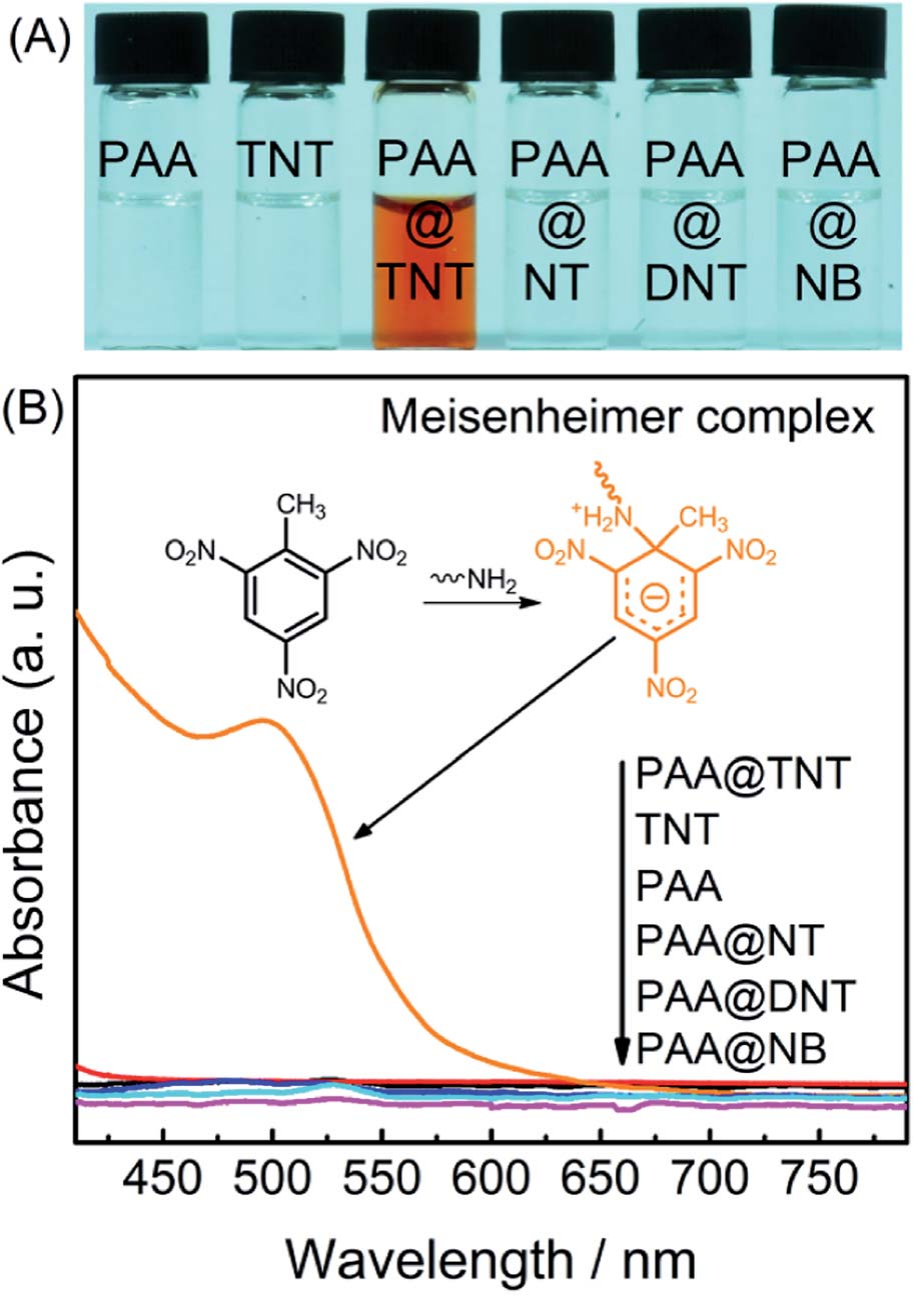
Characterization of the Meisenheimer complex between TNT and PAA: (A) photos of PAA, TNT, PAA–TNT, PAA–NT, and PAA–DNT solutions; and (B) the UV-vis absorption spectra of the above solutions. Concentration of PAA: 0.5 mg mL^–1^; concentration of TNT, NT, DNT, and NB: 400 μM.

After the successful identification of individuals with both green- and red-emitting QDs through LFP imaging, we investigated their potential in simultaneous visual detection of TNT in colloidal solution (the basis for TNT visualization in fingerprints). Selective interaction of TNT with primary amines has long been recognized for the development of various TNT sensors.^28^,^37^–^39^ Therefore, to bridge TNT with QDs and realize “traffic-light” type response for TNT, green QDs anchored onto the surface of red QD-doped silica nanoparticles were functionalized with PAA (Scheme 1). As an electron-withdrawing compound, TNT can readily react with an electron-donating primary amine to form the Meisenheimer complex, which often has a strong absorption in the visible range.^28^,^37^–^39^ Fig. 3 shows the UV-vis absorption spectra before and after adding PAA into TNT solution, which leads to a new visible absorption band at ∼505 nm (corresponding to the Meisenheimer complex between TNT and PAA). Meanwhile, one can clearly see that both TNT and PAA solutions are colorless, but a brown color was observed after mixing the two (Fig. 3A). Additionally, the absorption band of the Meisenheimer complex overlaps substantially with the fluorescence emission of the green-emitting QDs. Therefore, this Meisenheimer complex is expected to quench the fluorescence of green QDs through fluorescence resonance energy transfer (FRET, Fig. S6 of ESI†).^14^ Other TNT analogues, including 2,4-dinitrotoluene (DNT), nitrobenzene (NB) and paranitrotoluene (NT), did not cause a color change since they do not possess this specific interaction with primary amines.

**Fig. 4:**
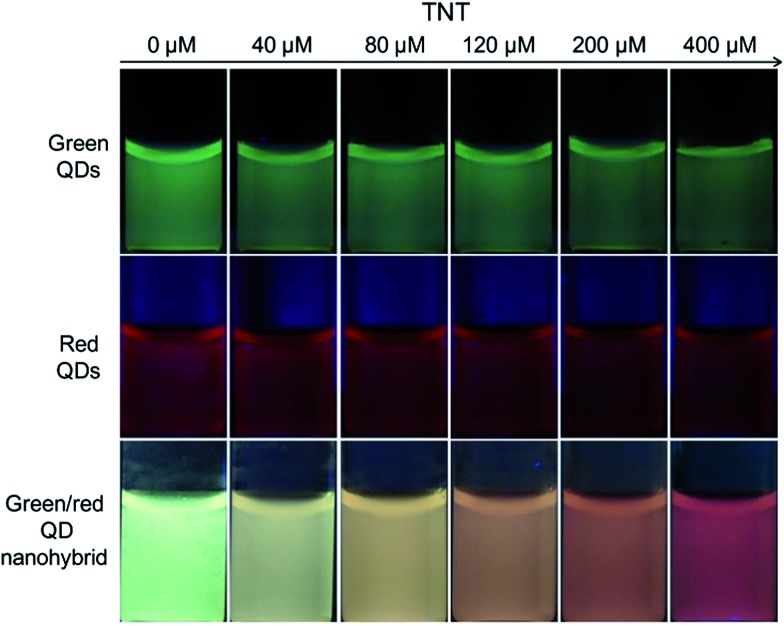
Photographs of the fluorescence images of green QDs, red QDs, and the green/red QD nanohybrid exposed to increasing amounts of TNT.

After being functionalized with PAA to provide a primary amine, the fluorescence intensity of the green PAA–ZnCdS QDs could be gradually quenched upon the addition of TNT (Fig. S7 of ESI†) due to the FRET from the QDs to the resultant Meisenheimer complex. The detection limit of TNT in solution was estimated to be about 5 μM from Fig. S7C (ESI†). However, the discrimination resolution of the fluorescence images was rather low and hard to distinguish among the other images by the naked eye (Fig. 4), since the human eye is not sensitive to a single color change. The red fluorescent color of the Cu–ZnCdS QD-doped silica nanoparticles remained almost unchanged, since there is no spectral overlap between the red QDs and the Meisenheimer complex. Therefore, the red fluorescent color can be explored as the reference to improve the discrimination resolution of green QDs for TNT visualization. After hybridizing the green and red QDs, the decrease of the green emission of the nanohybrid results in a gradual fluorescence color change of the nanohybrid from green to red when exposed to increasing amounts of TNT (Fig. 4), facilitating traffic light-type visual detection of TNT. This fluorescence color change in aqueous suspensions forms the basis for further visualization of TNT in fingerprints.

The nanohybrid exhibited reasonable selectivity for TNT over other explosives including DNT, NB, and NT. As shown in Fig. 3, only TNT can form the Meisenheimer complex with PAA, and the others cannot. Accordingly, only TNT can trigger the FRET between the green QDs and the Meisenheimer complex. Fluorescence investigations also confirmed this expectation, *i.e.*, DNT, NB, and NT showed much less quenching effect on the fluorescence intensity of PAA–green QDs, red QD, and the green/red QD nanohybrid (Fig. S8–S11 of ESI†). This selectivity feature can reduce the potential for false positives in fingerprint investigations.

### Exploration of the nanohybrid for simultaneous individual identification and TNT visualization

**Fig. 5:**
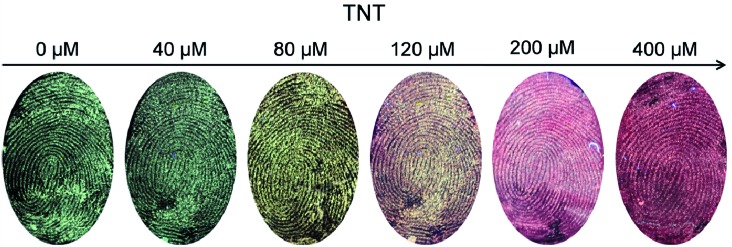
Imaging of LFPs with increasing amounts of TNT with the green/red QD nanohybrid: besides the ridge pattern of the fingerprint, the images also showed green to red color change (traffic light-type) with increasing amounts of TNT. All fluorescence images were excited with a 365 nm UV lamp.

During transportation or carrying of explosives, criminal suspects or terrorists will try to disguise themselves by posing as ordinary people. However, even with extremely cautious handling, TNT will adhere to their fingerprints after touching it. Therefore, a fingerprint can be an important type of evidence for police to build a connection between TNT and the carrier's identity, and identification and visualization of TNT in fingerprints is crucial for security-screening needs. For this purpose, a fingertip from the volunteer was first dipped into TNT solutions of six different concentrations, and then the fingerprints were collected and subjected to imaging. As shown in Fig. 5, all six LFPs were successfully stained with the developed nanohybrid. Meanwhile, the fluorescence color of the fingerprint images changed from green to yellow and then to red, which is consistent with the color change in the aqueous suspension (Fig. 4) and also with the traffic light change (green-yellow-red). The minimum detection limit of TNT in fingerprints was estimated to be 40 μM, which is higher than that in solution (5 μM). Moreover, all the obtained images showed well-resolved ridge flow and pattern configuration of the fingerprints (level 1 information). The level 2 characteristics of the fingerprint (ridge termination, bifurcation, and lake) could also be extracted from these images (Fig. S12 of ESI†) from the same place. Therefore, the fingerprint pattern can be used for individual identification and the image color can be used for visual detection of TNT.

To investigate the versatility of the nanohybrid for simultaneous individual identification and visual detection of TNT, LFPs deposited on several other nonporous surfaces of potential forensic interest were investigated. As shown in Fig. S13 of the ESI,† fingerprint images can also be taken from glass and paper. The image color of the LFPs with TNT on these surfaces is also different from that of the LFPs without TNT, demonstrating the robustness of the nanohybrid for identification and TNT visualization on a broad range of substrates.

## Conclusion

In summary, a nanohybrid composed of green- and red-emitting QDs was developed for latent fingerprint imaging. Through proper structural design, the nanohybrid also exhibited traffic light-type fluorescence color change when exposed to TNT. Thus, the nanohybrid could be used for both the identification of individuals and visual detection of TNT. We demonstrated the robustness of this nanohybrid for imaging of both fingerprints and TNT on several other substrates. This method is promising for potential applications in security-screening needs.

## Experimental

### Materials

Cadmium nitrate (Cd(NO_3_)_2_, AR), zinc nitrate (Zn(NO_3_)_2_, AR), sodium sulfide (Na_2_S, AR), sodium hydroxide (NaOH, AR), silver nitrate (AgNO_3_, AR) and copper acetate (Cu(Ac)_2_, AR) were purchased from Chengdu Kelong Chemical Reagent Company (Chengdu, China). 3-Mercaptopropionic acid (MPA, 98%), l-cysteine (l-Cys, 99%), (3-aminopropyl)-triethoxysilane (APTS, 99%), 3-mercaptopropyltrimethoxysilane (MPS), ammonia solution (NH_3_·H_2_O, GR 25–28%), tetraethyl orthosilicate (TEOS, 99.99%), polyallylamine (PAA, *M*_w_ ≈ 65 000), 1-(3-dimethylaminopropyl)-3-ethylcarbodiimide hydrochloride (EDC), and *N*-hydroxysuccinimide (NHS) were obtained from Aladdin (Shanghai, China). 2,4,6-Trinitrotoluene (TNT), 2,4-dinitrotoluene (DNT), nitrobenzene (NB) and paranitrotoluene (NT) were kindly supplied by Dr Wen-Sheng Zou (Anhui University of Architecture). All chemicals were used directly without further purification. Caution: the highly explosive TNT should be used with extreme caution and handled only in small quantities.

### Synthesis and characterization of QDs and QD assemblies

The syntheses of both green- and red-emitting QDs were based on a previous report of the synthesis of doped ZnCdS QDs in a high-temperature organic phase.^40^ However, we revised the synthetic protocol from an organic to an aqueous phase. The detailed synthesis is given in the ESI.† For preparation of the red QDs@silica@green QD nanohybrid, a synthetic protocol by Zhang *et al.*^28^ was employed (Scheme 1 and ESI†). Red-emitting Cu–CdZnS QDs were first incorporated into silica with the well-known Stöber method (red QD-doped silica). Next, green-emitting CdZnS QDs were anchored onto the surface of silica assisted by EDC/NHS coupling and further functionalized with PAA (as receptor for TNT).

The UV-vis and fluorescence emission spectra of the QDs were obtained with a UV-1700 UV/Vis spectrophotometer (Shimadzu, Japan) and an F-7000 spectro-fluorometer (Hitachi, Japan), respectively. TEM and EDX characterization was performed with a Tecnai G2 F20 S-TWIN transmission electron microscope at an accelerating voltage of 200 kV (FEI Co., America). Samples for TEM were prepared by dropping a dilute solution of NPs onto nickel grids (for the Cu-doped QDs) or copper grids (for the ZnCdS QDs) with subsequent evaporation of the solvent in air at room temperature.

### Protocol for fingerprint acquisition

All sebaceous fingerprints were acquired from the same volunteer. The hands were thoroughly washed with soap before fingerprint deposition. The fingertip was first dipped into TNT solutions of different concentrations and then gently rubbed across the forehead before stamping the finger onto the substrate, such as aluminum foil mainly. The foil strips bearing the fingerprints were immersed in the aqueous solution of dual-emission doped QDs for 30 s and taken out. The fingerprint images under UV lamp irradiation (365 nm) and other fluorescence images were photographed with a Nikon D300S digital camera equipping a Nikon AF-S VR 105mm f/2.8G IF-ED Macro Lens without any filters.

## Supplementary Material

Supplementary information
