# Supplementary material for: Dual-emitting quantum dot nanohybrid for imaging of latent fingerprints: simultaneous identification of individuals and traffic light-type visualization of TNT
Source: Chem Sci. 2015 May 21;6(8):4445–50. doi: 10.1039/c5sc01497b (PMC6088367; doi:10.1039/c5sc01497b)
Supplement: Supplementary file 1 [file SC-006-C5SC01497B-s001.pdf]

Electronic Supplementary Material (ESI) for Chemical Science.
This journal is © The Royal Society of Chemistry 2015

Electronic Supplementary Information

Dual-Emitting Quantum Dots Nanohybrid for Imaging of Latent Fingerprints: Simultaneous Identification of Individuals and Traffic Light-Type Visualization of TNT

Peng Wu,[†,‡] Chaoying Xu,[‡] Xiandeng Hou,[‡] Jing-Juan Xu,[*,†] Hong-Yuan Chen,[*,†]

[†]State Key Laboratory of Analytical Chemistry for Life Science and Collaborative Innovation Center of Chemistry for Life Sciences, School of Chemistry and Chemical Engineering, Nanjing University, Nanjing 210093, China

[‡]Analytical & Testing Center, Sichuan University, Chengdu 610064, China

*Corresponding author, E-mail: xujj@nju.edu.cn, hychen@nju.edu.cn

Preparation of ZnCdS QDs

For ZnCdS QDs, $Zn(NO_3)_2$ (0.05 mL, 0.2 M), $Cd(NO_3)_2$ (0.8 mL, 0.4 M), and L-Cys (0.08 g) were mixed with 20 mL ultrapure water in a four-neck round-bottomed flask. The pH of the mixed solution was adjusted to ~11 with 2.0 M NaOH. Freshly prepared Na_2S (0.2 mL, 0.4 M) was quickly injected with stirring for 2 min. Then, the mixture solution was refluxed at 60 ºC for 2 min. Afterwards, $Zn(NO_3)_2$ (0.08 mL, 0.2 M) was injected into the reaction solution, and refluxed at 100 ºC for another 2 min. The obtained green (ZnCdS) QDs were precipitated with ethanol, and separated by centrifugation. The resultant QDs were directly dissolved in water for further use.

Preparation of Cu-doped ZnCdS QDs

For Cu-doped ZnCdS QDs, $Zn(NO_3)_2$ (0.05 mL, 0.2 M), $Cd(NO_3)_2$ (0.8 mL, 0.4 M), $Cu(Ac)_2$ (0.082 mL, 0.02 M), and MPA (0.056 mL) were combined with 40 mL ultrapure water in a four-neck round-bottomed flask. The pH of the mixed solution was adjusted to ~11 with 2.0 M NaOH. Freshly prepared Na_2S (0.8 mL, 0.4 M) was quickly injected with stirring for 10 min. Then, the mixture solution was refluxed at 100 ºC for 2 h. Afterwards, $Zn(NO_3)_2$ (0.08 mL, 0.2 M) was injected into the reaction solution, and refluxed at 100 ºC for another 10 min. The obtained MPA-capped Red (Cu-doped ZnCdS) QDs were precipitated with ethanol, and separated by centrifugation. The resultant QDs were directly dissolved in water for further use.

Preparation of red fluorescent QD-doped silica nanoparticles

The red fluorescent QDs incorporated silica nanoparticles were synthesized by a modified Stöber method.[S1] Briefly, ethanol (20 mL), MPS (10 μL), red-emitting Cu-ZnCdS QDs (10 mL, pH 11) were mixed under stirring in a 100 mL three-necked flask for 6 h. Then, 0.2 mL of TEOS and 0.2 mL of ammonium hydroxide were added, and the mixture was stirred for 12 h. To modify the silica surface with amino groups, 10 μL of APTS was added to the above solution and stirred for another 12 h. After the reaction, the as-prepared nanoparticles were washed with ethanol and ultrapure water for several times to remove the un-reacted chemicals.

Synthesis of dual-emitting red QDs@silica@green QDs nanohybrid

4 mL green QDs solution and 4 mL red QD-doped silica nanoparticles were dispersed in 4 mL ultrapure water, and then 2 mL of EDC/NHS (2 mg/mL) was added. The final mixture was stirred for 2 h at room temperature in the dark. The resulting nanohybrid of red QDs@silica@green QDs were isolated by centrifugation and washed with ultrapure water to remove the excess QDs and chemicals. The nanohybrid was further modified in the mixed solution of 0.5 mg polyallylamine, 1 mL EDC/NHS (2 mg/mL) and 8 mL ultrapure water by shaking for 0.5 h. The obtained nanohybrid was washed with ultrapure water and re-dispersed in 5 mL ultrapure water.

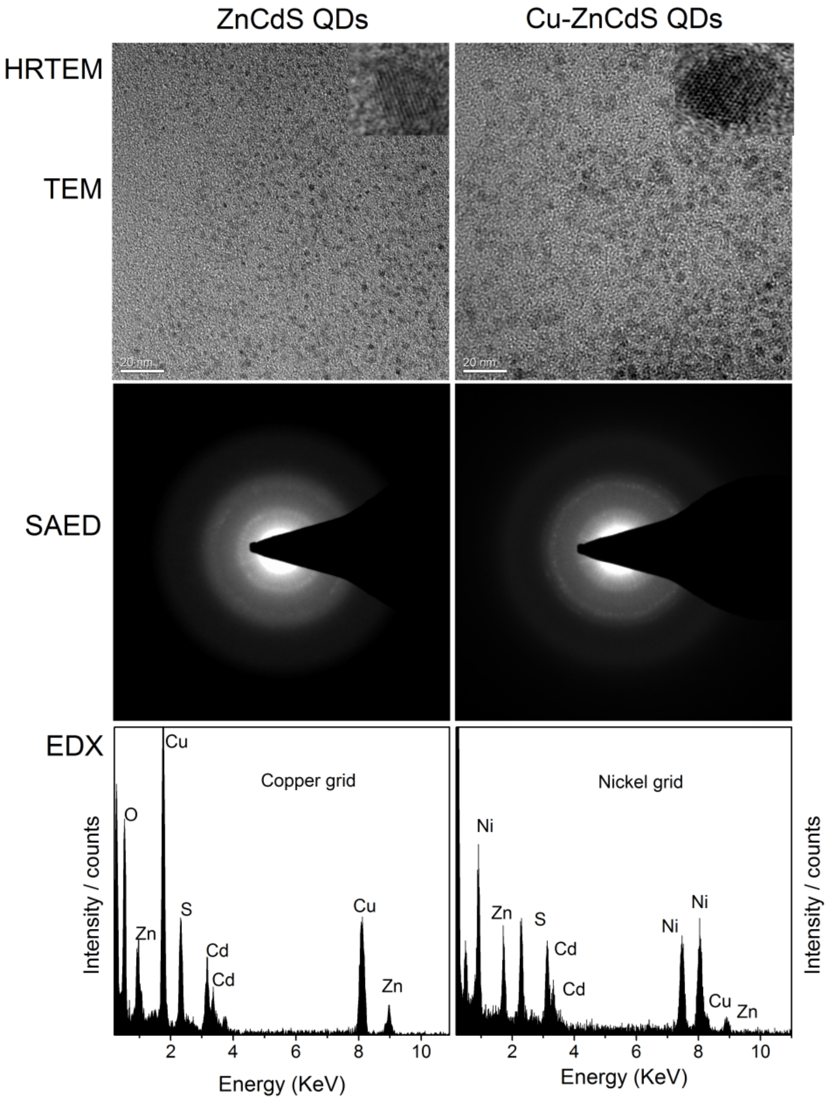

Figure S1. Characterization of ZnCdS and Cu-doped ZnCdS QDs with TEM, HRTEM, selected-area electron diffraction (SAED), and Energy dispersive X-ray analysis (EDX).

Table S1. Elemental composition of ZnCdS and Cu-ZnCdS QDs from ICP-OES analysis.

	Zn	Cd	Cu	Total
ZnCdS QDs	8.6%	91.4%	-	100%
Cu-ZnCdS QDs	2%	97%	1%	100%

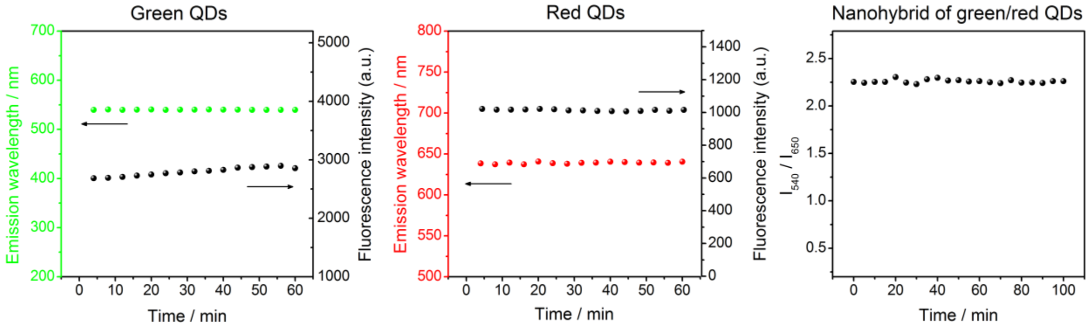

Figure S2. Photostability investigation of green, red, and nanohybrid of green/red QDs under continuously irradiated with 365 nm.

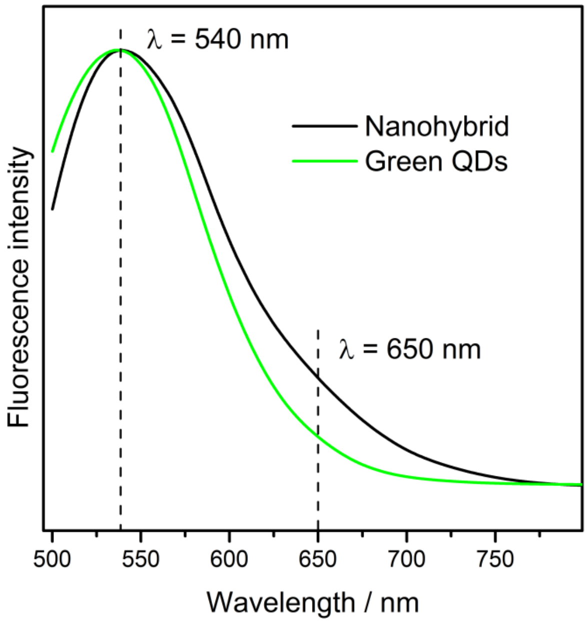

Figure S3. Comparison of the fluorescence spectra of green QDs and nanohybrid excited with 365 nm. The convex at about 650 nm of the nanohybrid was the contribution from red-emitting QDs.

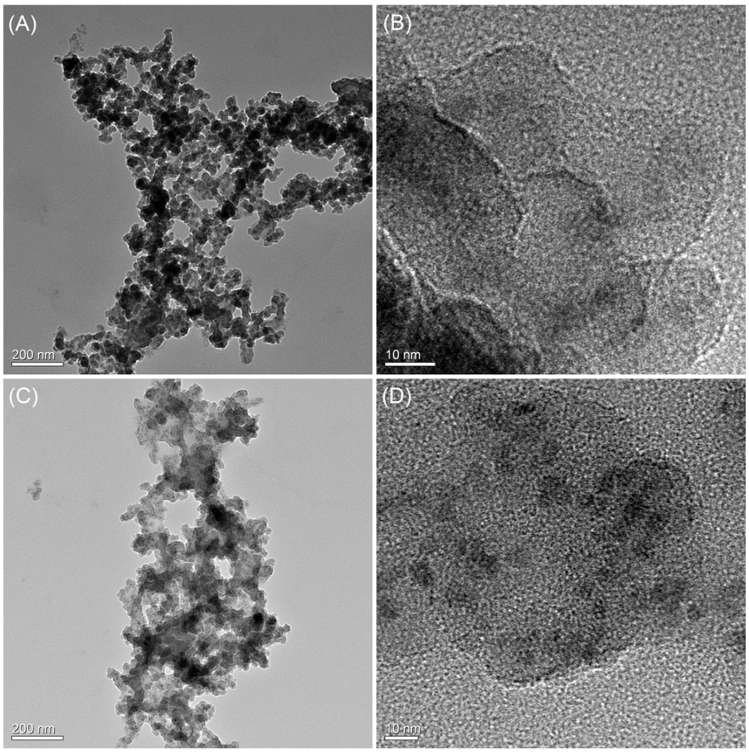

Figure S4. TEM (A, C) and HRTEM (B, D) characterization of nanohybrid: (A) and (B) red QDs-doped silica nanoparticles; (C) and (D) nanohybrid of red QDs@silica@green QDs.

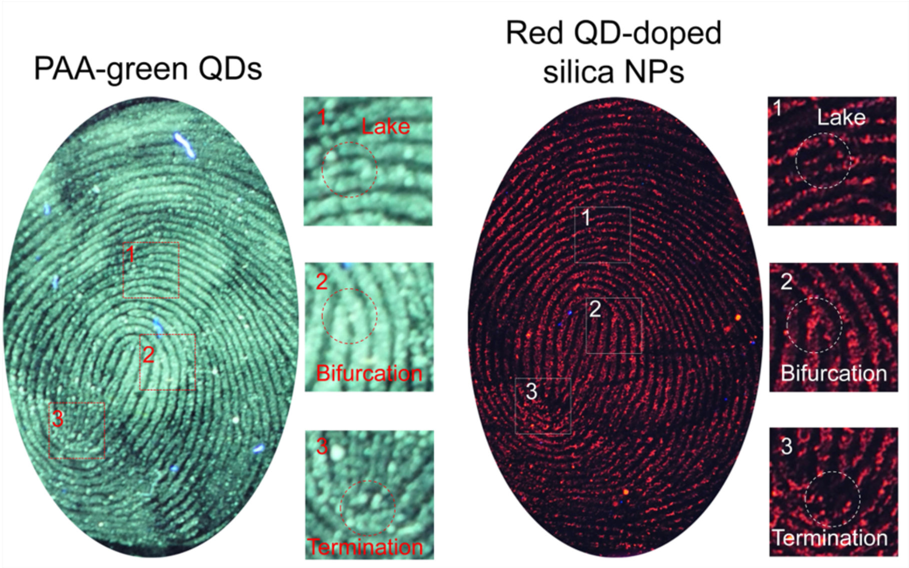

Figure S5. LFPs stained with green and red QDs and the details of level 2 characteristics (lake, bifurcation, and termination). All fluorescent images were excited with a 365 nm UV lamp.

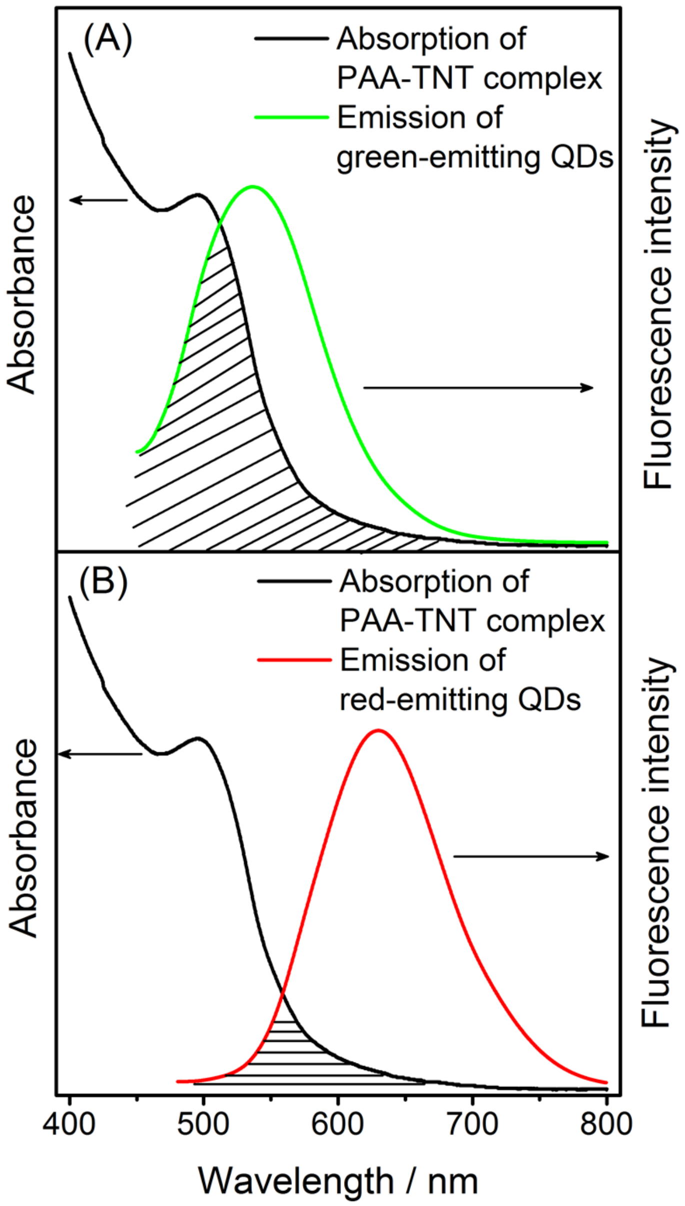

Figure S6. Overlap between the absorption of TNT-PAA Meisenheimer complex and the emission profile of green-emitting QDs (A) and red-emitting QDs (B). It's clear that the overlapped part between TNT-PAA Meisenheimer complex and the emission profile of green-emitting QDs is obviously larger than that of red-emitting QDs.

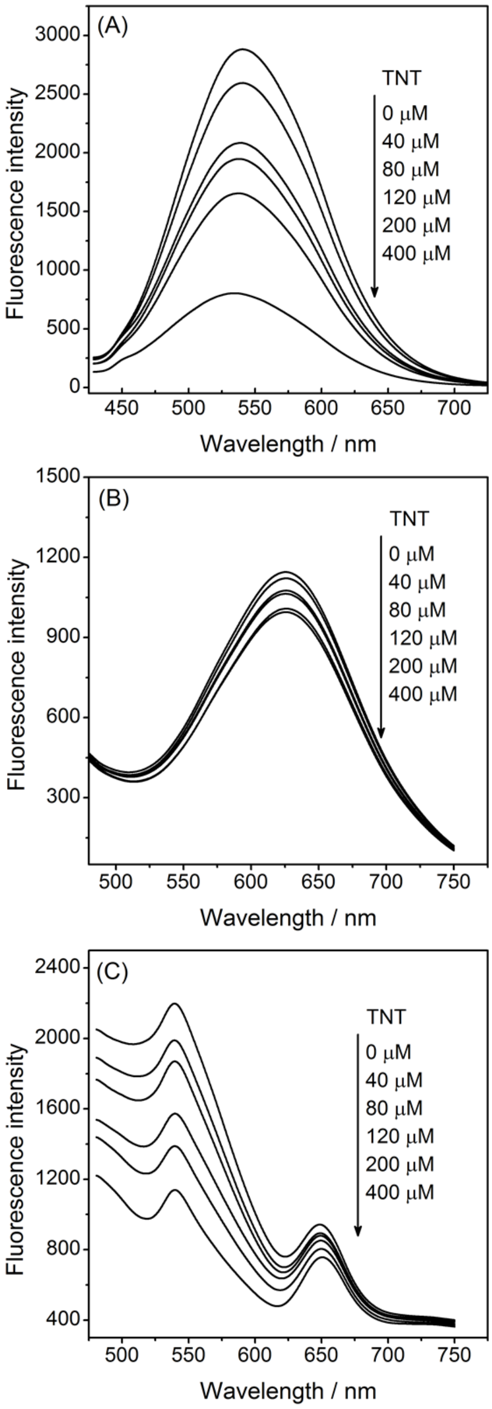

Figure S7. Fluorescence emission spectra (λ_{ex} = 425 nm) of green QDs (A), red QDs-doped silica nanoparticles (B), and nanohyrbrid of green/red QDs (C) in the presence of increasing amounts of TNT. The detection limit of TNT in solution was estimated to be about 5 μM from Figure S7C. The intensity decrease of red QDs in Figure S7C was largely because of the large FWHM of green-emitting QDs that resuted in cross-talking to the red QD reference.

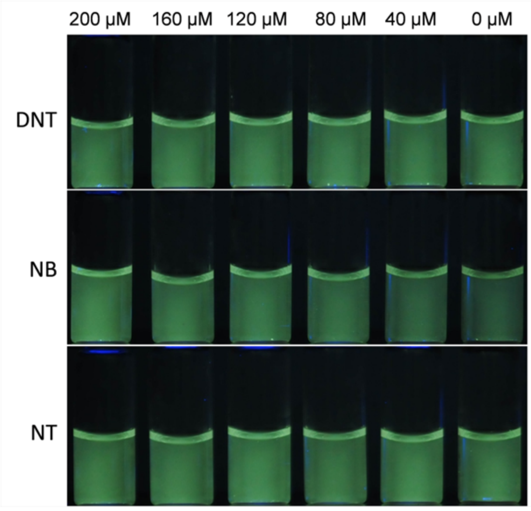

Figure S8. Photos of the fluorescent images of green QDs, challenging with increased amounts of DNT, NB and NT.

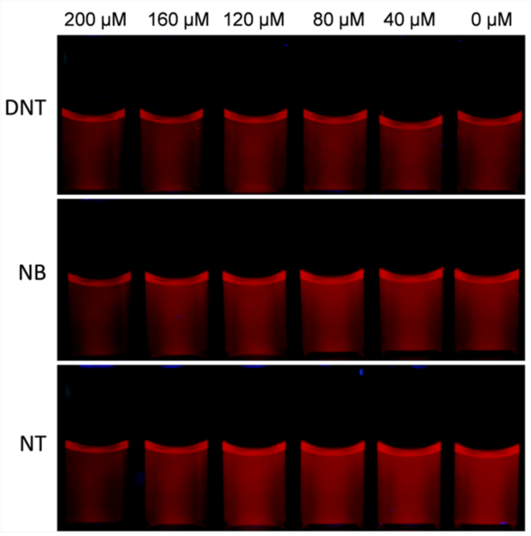

Figure S9. Photos of the fluorescent images of red QDs, challenging with increased amounts of DNT, NB and NT.

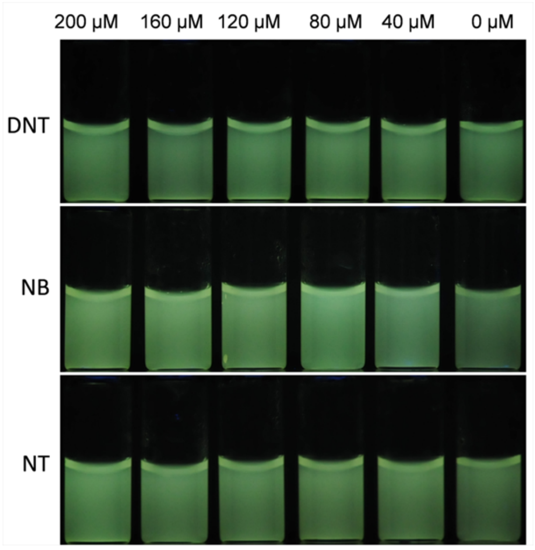

Figure S10. Photos of the fluorescent images of nanohybrid, challenging with increased amounts of DNT, NB and NT.

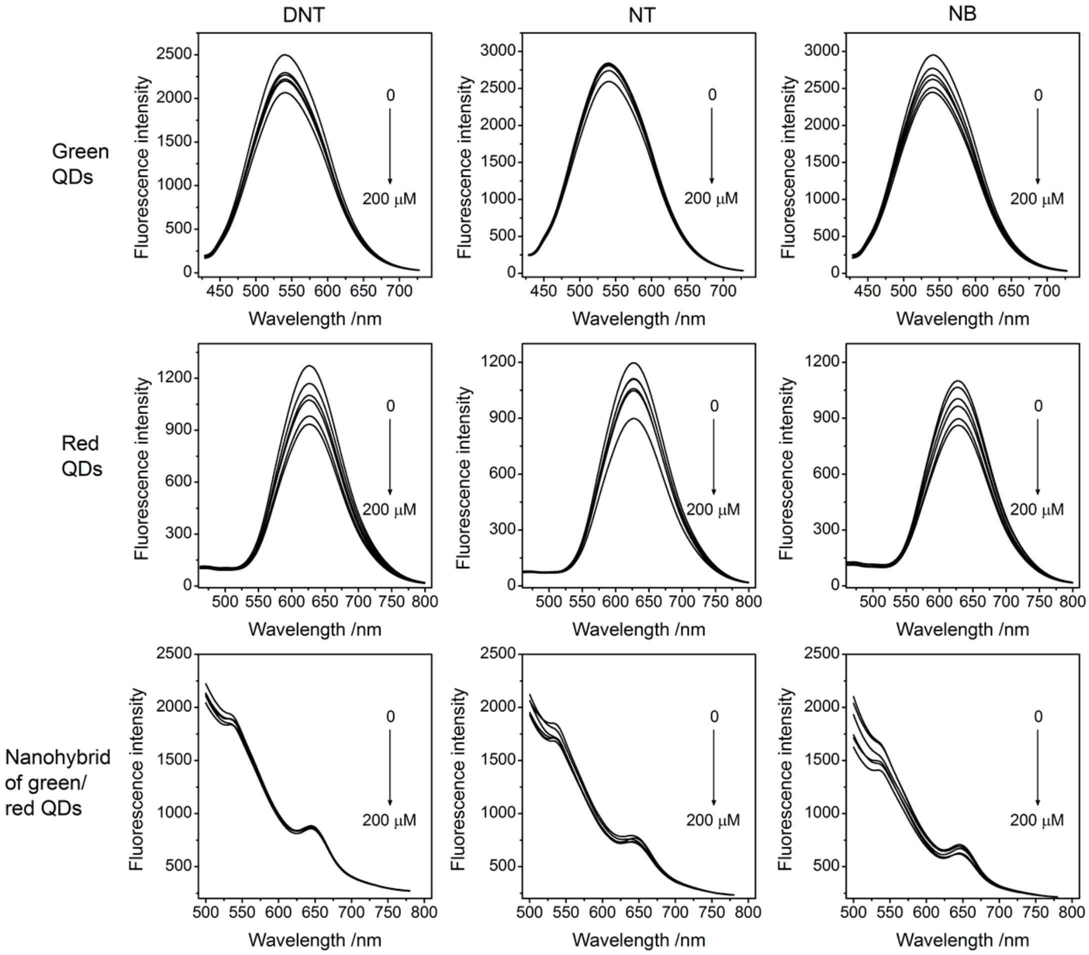

Figure S11. Fluorescence emission spectra (λ_{ex} = 425 nm) of green QDs, red QDs, and nanohyrbrid of green/red QDsin the presence of increasing amounts of DNT, NT, and NB.

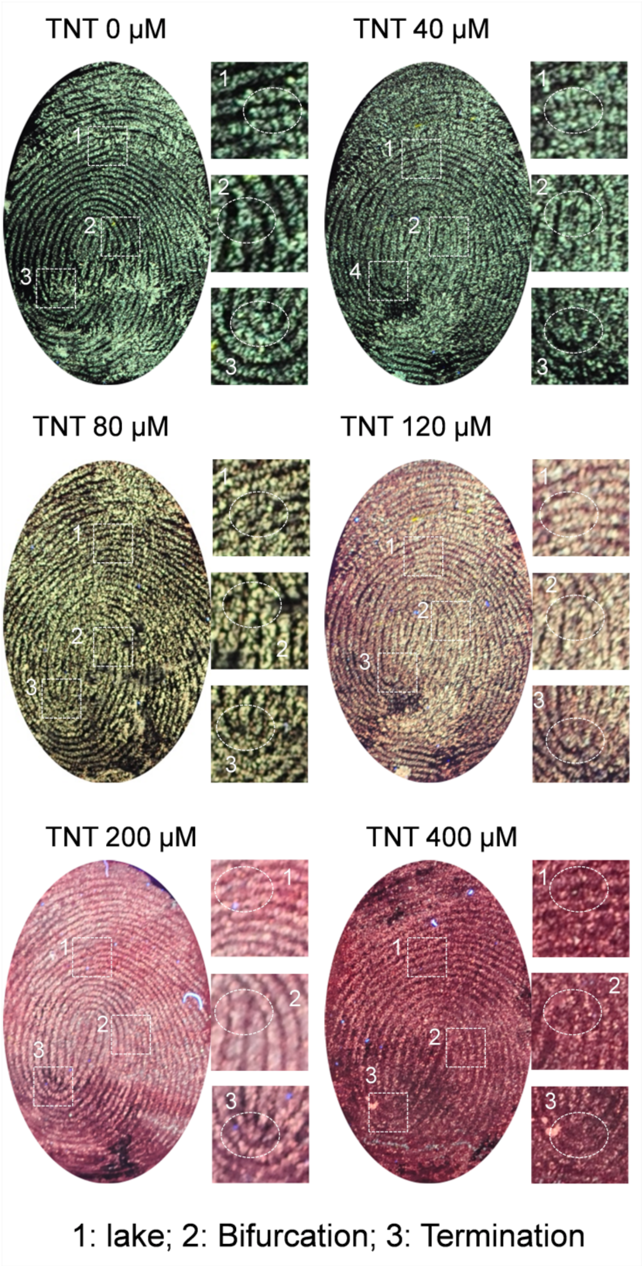

Figure S12. LFPs with different amounts of TNT stained with the green/red QDs nanohybrid and the details of level 2 characteristics (lake, bifurcation, and termination). All fluorescent images were excited with a 365 nm UV lamp.

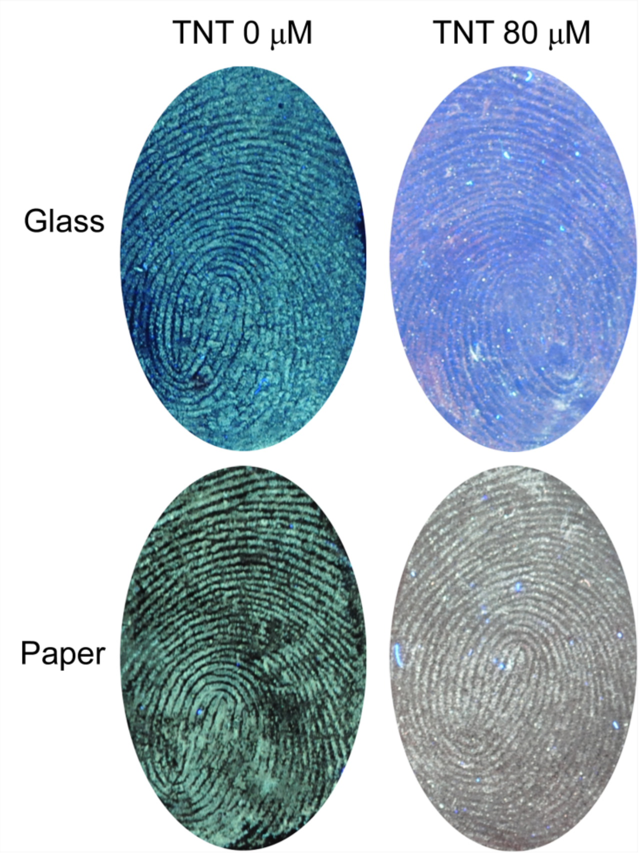

Figure S13. Imaging of LFPs with or without TNT on glass and paper substrates with the green/red QDs nanohybrid. Note, the image color of LFP on these substrates is different from that on aluminum foil strips (in the text), because these substrates have background fluorescence. All fluorescent images were excited with a 365 nm UV lamp.

References

[S1] Zhang, K.; Zhou, H. B.; Mei, Q. S.; Wang, S. H.; Guan, G. J.; Liu, R. Y.; Zhang, J.; Zhang, Z. P. *J. Am. Chem. Soc.* **2011**, *133*, 8424-8427.
